# Regulatory roles of alternative splicing at *Ezh2* gene in mouse oocytes

**DOI:** 10.1186/s12958-022-00962-x

**Published:** 2022-07-05

**Authors:** Shi-meng Guo, Xing-ping Liu, Qing Tian, Cai-feng Fei, Yi-ran Zhang, Zhi-ming Li, Ying Yin, Ximiao He, Li-quan Zhou

**Affiliations:** 1 Institute of Reproductive Health, Tongji Medical College, Huazhong University of Science and Technology, Wuhan, Hubei China; 2 School of Basic Medicine, Tongji Medical College, Huazhong University of Science and Technology, Wuhan, Hubei China

**Keywords:** *Ezh2*, Alternative splicing, Oocyte, Mitochondria

## Abstract

**Background:**

Enhancer of zeste homologue 2 (EZH2), the core member of polycomb repressive complex 2 (PRC2), has multiple splicing modes and performs various physiological functions. However, function and mechanism of alternative splicing at *Ezh2* exon 3 in reproduction are unknown.

**Methods:**

We generated *Ezh2*^Long^ and *Ezh2*^Short^ mouse models with different point mutations at the *Ezh2* exon 3 alternative splicing site, and each mutant mouse model expressed either the long or the short isoform of *Ezh2*. We examined mutant mouse fertility and oocyte development to assess the function of *Ezh2* alternative splicing at exon 3 in the reproductive system.

**Results:**

We found that *Ezh2*^Long^ female mice had normal fertility. However, *Ezh2*^Short^ female mice had significantly decreased fertility and obstructed oogenesis, with compromised mitochondrial function in *Ezh2*^Short^ oocytes. Interestingly, increased EZH2 protein abundance and accumulated H3K27me3 were observed in *Ezh2*^Short^ oocytes.

**Conclusions:**

Our results demonstrate that correct *Ezh2* alternative splicing at exon 3 is important for mouse oogenesis.

**Supplementary Information:**

The online version contains supplementary material available at 10.1186/s12958-022-00962-x.

## Background

Alternative splicing of pre-mRNA is an essential post-transcriptional mechanism to maintain coordinated gene expression in the mammalian germline. mRNA precursors produce different mRNA splicing isoforms through various splicing events, and these isoforms may differ in transcript stability, translation activity and protein stability, and may have distinct regulatory effects on development and organ formation [1]. In physiological conditions, 90% of human genes and 63% of mouse genes can be alternatively spliced [4, 6]. Accumulated evidence has shown that alternative splicing is a key step in gene expression cascade, which greatly affects the diversification and spatiotemporal control of the proteome in multiple developmental processes [18].

Enhancer of zeste homologue 2 (EZH2) is a core member of the polycomb repressive complex 2 (PRC2), which catalyzes H3K27me3 modification through the SET (suppressor of variegation, enhancer of zeste and trithorax) domain [2]. In the central nervous system, loss of the *Ezh2* splice variant X9 significantly impaired the proliferation and differentiation of neurons [12]. Alternative splicing has also been widely reported in the reproductive system [17]. After conditional knockout of *Ezh2* in mouse oocyte, the level of H3K27me3 in the oocyte was significantly reduced. Although early embryonic development proceeded normally, offspring weight was reduced [7]. A recent report also indicated that maternal EZH2 is necessary to establish H3K27me3 in mouse zygotes [16].

*Ezh2* is critical in X chromosome inactivation and the embryonic lethality of *Ezh2* knockout mice indicates that it is essential in early development [21]. The lack of *Ezh2* also blocks developmental potency of embryonic stem cells and the differentiation of trophectoderm cells [9, 21]. Multiple alternative splicing products of *Ezh2* may be responsible for orchestrating various cellular events in development and stress conditions [8, 20]. Different isoforms of *Ezh2* have been reported in testicular tissues, with exon 14 deletion and exon 3 truncation more commonly identified. The short isoform with deletion of exon 14 only exists in primary spermatocytes and has a regulatory role in the process of spermatocyte meiosis [20]. However, the function and mechanism of *Ezh2* exon 3 truncation is unknown.

In this study, we found that alternative splicing sites in the *Ezh2* locus located at the 3' end of exon 3 and the 5' end of exon 4 were conserved between mouse and human. Next, we engineered mice with point mutations to express only the long (*Ezh2*^Long^) or the short isoform (*Ezh2*^Short^), and identified disturbed female reproduction induced by changes of the alternative splicing event. Further investigations showed that expression of only short isoform of *Ezh2* led to decline of female fertility and disturbance of mitochondrial functions in oocytes.

## Methods

### Mice

The mice used in this study had C57BL/6 background. All mice were housed in SPF-level experimental animal centers free of special pathogens. Mutant mice were generated by GemPharmatech (Nanjing, China) and CRISPR-Cas9 homologous recombination technology was used to generate *Ezh2*^Long^ and *Ezh2*^Short^ mice. Generally, the constitutive Cas9 mRNA, sgRNA (long only: 5’-ACTTCTGTGAGCTCATTGCGCGG-3’, short only: 5’-TTGCGCGGGACTAGGGAGGTTGG-3’) and donor oligos (long only: 5’-AAACCAAGAGTGGAAGCAGCGGAGGATACAGCCTGTGCACATCATGACTTCTCTGAGCTCATTGAGAGGGACTAGGGAGGTTGGTTAACACAGTGTTGCAACAATTCTCAACACATTTGC-3’, short only: 5’-CGGAGGATACAGCCTGTGCACATCATGACTTCTGTGAGCTCATTGCGCGGGACTAGGGAGCTTGGTTAACACAGTGTTGCAACAATTCTCAACACATTTGCTTTCTACTTATTGTAATGT-3’) were co-injected into zygotes with standard microinjection setup, in order to create a DSB which was further repaired by donor-mediated homology directed repair (HDR) to introduce mutations into *Ezh*2 locus.

### Genotyping

Total genomic DNA was extracted from mouse tails for lysis. PCR was performed for 30 cycles at 95 °C for 30 s, 60 °C for 30 s, and 72 °C for 30 s, with a final extension at 72 °C for 5 min. Two pairs of primers were used simultaneously in this experiment, *Ezh2*^Long^ (*Ezh2*^Long^_1_Forward: 5’-AACCGGCTTTAAAGATGCTACATATTGTAACA-3’, *Ezh2*^Long^_1_Reverse: 5’-ACATTACAATAAGTAGAAAGCAAATGTGTTGAGAAT-3’, *Ezh2*^Long^_2_ Forward: 5’-CTAGTCCCGCGCAATGAGCTCAC-3’, *Ezh2*^Long^_2_Reverse: 5’-ATACAGCCTGTGCACATCATGACTTCTC-3’), *Ezh2*^Short^ (*Ezh2*^Short^_1_Forward: 5’-GAGTGTGGAGAATTGTAGTATCTTGTGTGAATCTGT-3’, *Ezh2*^Short^_1_ Reverse: 5’-GAGAGACATCCTGAAGTCTGTGAATTCTTC-3’, *Ezh2*^Short^_2_Forward: 5’-TTGAGAATTGTTGCAACACTGTGTTAACCAAC-3’, *Ezh2*^Short^_2_ Reverse: 5’-ATTGCGCGGGACTAGGGAGC-3’). PCR products were examined by Sanger sequencing in Tsingke Biotechnology company (Wuhan, China). The sequencing results were analyzed using SnapGene software.

### Fertility assessment and testis/ ovarian histology analysis

The adult homozygous mutant (*Ezh2*^Long^ or *Ezh2*^Short^) male/female mice and adult wild-type female/male mice were mated in one cage at a ratio of 1:1 as the experimental group, with mating of adult wild-type male and female mice served as the control group. There were 5 mice in each of the experimental groups and the control group for the fertility test. The observation period was 6 months, and the average litter size was analyzed and compared. For histology analysis, testis/ovarian sections were prepared for hematoxylin–eosin staining as before [25]. Tissues were collected from 3 male/female mice separately.

### Oocytes collection and culture

Follicles were pierced from ovaries of experimental group (2-month-old mice) and the control group (2-month-old mice) with an injection needle to release the oocytes, and the intact GV oocytes which were naturally without cumulus cells were transferred to clean M2 medium (M1250, Aibei, China) separately with mineral oil at 37 °C in a 5% CO_2_ incubator. In order to obtain MII oocytes, female mice were injected with 10 IU of pregnant mares serum gonadotropin (PMSG, 110,914,564, SANSHENG, China), followed by injection of 10 IU of human chorionic gonadotropin (HCG, 110,911,282, SANSHENG, China) 48 h later. MII oocytes were obtained from the oviducts after 16 h of HCG injection and transferred to clean M2 medium for later use.

### TUNEL assay

TUNEL assay was performed in ovary paraffin sections according to the instructions provided by the TUNEL BrightGreen Apoptosis Detection Kit (A112, Vazyme, China). Fluorescence was detected using Mshot Inverted Fluorescence Microscope.

### Immunofluorescence

Oocytes were fixed with 4% paraformaldehyde (E672002, Sangon Biotech, China) for 30 min at room temperature, then they were permeabilized in 1% (vol/vol) Triton X-100 (A110694, Sangon Biotech, China) for 20 min. After blockage at 37 °C for 1 h with 1% (wt/vol) bovine serum albumin (BSA, A600903, Sangon Biotech, China), the oocytes or embryos were incubated with primary antibodies against TOM20 (1:100, A19403, Abclonal Biotech, China), EZH2 (1:100, ab191080, Abcam, UK), H3K27me3 (1:100, ab6002, Abcam, UK), H3K4me3 (1:200, 9727, CST, USA), H3K9me3 (1:200, 13,969, CST, USA) and H3K9ac (1:200, A7255, Abclonal Biotech, China) at 4 °C overnight. The corresponding secondary antibodies CoraLite594-conjugated Goat Anti-Rabbit IgG (H + L) (1:400, SA00013-4, Proteintech Group, Inc, USA), were added and incubated at 37 °C for 2 h. Samples were observed under LSM 780 confocal microscope. Immunofluorescence intensity levels were assessed by Image J software. After adding the image, a single channel was extracted and converted to 8-bit format. The software default threshold was used and measuring parameters were specified (Area (area of the selected area), Mean (average gray value of the selected area), Integrated Density (total fluorescence intensity of the selected area)). Each area was selected by lasso tool and circumscribed by “ROI Manager” function, and “Measure” command was used to obtain output data.

### Mitochondrial distribution assay

Mito-Tracker Red CMXRos (C1035, Beyotime, China) was used to detect mitochondrial distribution. Live oocytes were cultured in M2 medium containing 200 nM Mito-Tracker Red CMXRos for 30 min at 37 °C. Before examining, the nuclei were stained with Hoechst 33,342 (10 μg/ml, C1022, Beyotime, China).

### Mitochondrial membrane potential (MMP) examination

Live oocytes were incubated in M2 medium with 10 μg/ml JC-1 (C2006, Beyotime, China) for 30 min at 37 °C and then washed three times. Samples were observed under stereoscopic microscope (Mingmei, China). Immunofluorescence intensity levels were assessed by Image J software (mentioned above).

### mtDNA copy number measurements

Pool of 15 oocytes were transferred to 10 µl lysis buffer (50 mM Tris–HCl, 200 μg/ml proteinase K, 0.5%Triton X-100) at 55 °C for 2 h. Real-time PCR was performed to measure mtDNA copy number. mtDNA primers (Forward: 5’-CTAGCAGAAACAAACCGGGC-3’, Reverse: 5’-CCGGCTGCGTATTCTACGTT-3’). Each experiment was repeated for at least three times independently. mtDNA levels were normalized to *β-actin* locus.

### ROS assessment

ROS levels of oocytes were determined by H2DCFDA (MedChemExpress, HY-D0940, USA). Generally, oocytes were incubated with 5 µM H2DCFDA solution for 30 min at 37 °C. After oocytes were washed twice in M2, they were quickly placed on a glass slide, and the fluorescence intensity of oocytes was immediately observed.

### Real-time RT-PCR

Total RNA was extracted from 50 oocytes using TRIzol reagent (Invitrogen, USA) following the manufacturer’s procedure and cDNA synthesis was completed using Hifair 1st Strand cDNA Synthesis Kit (Yeason, China). RT-PCR was performed with SYBR green master mix (Yeason, China) with ABI 7500 Real-Time PCR system (Applied Biosystems, USA). Mouse *Ezh2* (Forward: 5’-GAGTGGAAGCAGCGGAGGAT-3’, Reverse: 5’-TGTAAGGGCGACCAAGAGTA-3’), *Cps1* (Forward: 5’- TTCCCTCTGACTATGTTGCC-3’, Reverse: 5’- TTGAGCCAGTCTGATGTAGC-3’), *Yy1* (Forward: 5’- GGGATACCTGGCATTGACCT-3’, Reverse: 5’- CACTCTGCACAGACGTGGACT-3’), and *Xist* (Forward: 5’- TGGTTCGTCTATCTTGTGGGTC-3’, Reverse: 5’- CTGGGAGAACTGCTGTTGTGAT-3’), was normalized against mouse *β-actin* (Forward: 5’- GGCTGTATTCCCCTCCATCG -3’, Reverse: 5’- CCAGTTGGTAACAATGCCATGT -3’). Quantification of the fold change was determined by the comparative CT method.

### RNA-seq analysis

We collected 8–10 GV oocytes for each group in lysis component with ribonuclease inhibitor, and amplification was further carried out using the Smart-Seq2 method by Annoroad Gene Technology Corporation (Beijing, China). Qualified libraries were loaded onto Illumina Hiseq platform for PE150 sequencing. Raw reads were processed with cutadapt v1.16 to remove adapters and perform quality trimming with default parameters except for quality-cutoff 20, minimum-length 20. Trimmed reads were mapped to mouse genome (GENCODE release M23) using STAR with default settings. Reads were counted in exons of the mouse genome, using the STAR-quantMode GeneCounts setting. RSEM was used to calculate FPKM value. Differentially regulated genes in the DESeq2 analysis were defined as those which were more than two-fold increased or decreased with adjusted *P* < 0.05. Gene ontology (GO) analysis, Kyoto Encyclopedia of Genes and Genomes (KEGG) analysis were performed by Metascape (https://metascape.org). Volcano, heatmap, Bar chart and bubble chart were generated by R.

### Statistical analysis

Data were presented as mean ± SEM. All experiments were replicated more than three times. Statistical comparisons were made with Mann–Whitney U test for analysis of 2 groups. Kruskal–Wallis test with post-hoc analysis was used to analyze differences among 3 groups. Analyses were conducted using SPSS 20 software (IBM). *P*-value < 0.05 (*P < 0.05, **P < 0.01, ****P < 0.0001) was considered to be statistically significant.

## Results

### Alternative splicing sites of *Ezh2* at Exon 3 are conserved between mouse and human

**Fig. 1:**
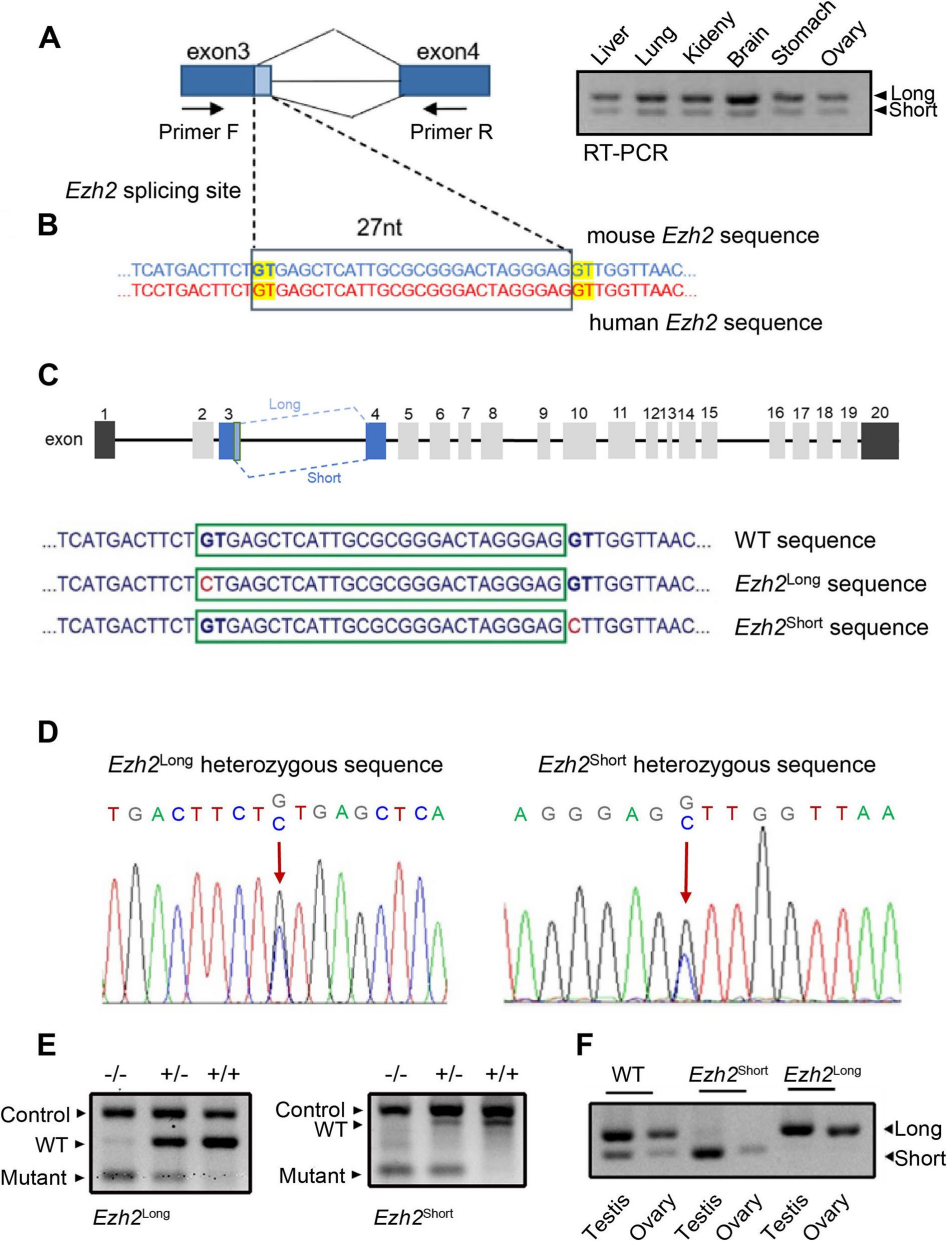
Identification of the alternative splicing site within the mouse *Ezh2* locus and the point mutation strategy. **A** Left panel shows alternative splicing sites and RT-PCR primer positions. Right panel shows RT-PCR result of the *Ezh2* alternative splicing event in multiple mouse tissues. **B** Conservation analysis of the alternative splicing site sequence at mouse/human *Ezh2* gene loci; the additional 27 nt DNA sequence in the long isoform encodes nine amino acids. The donor site sequence "GT" is marked in yellow. **C** WT mice expressed both the long isoform and the short isoform. *Ezh2*^Long^ expressed only the long isoform. *Ezh2*^Short^ expressed only the short isoform. Mutation strategy at *Ezh2* alternative splicing site: mutation of the donor site sequence "GT" (the base marked in red is the sequence after mutation) to produce only the long isoform (*Ezh2*^Long^) or the short isoform (*Ezh2*^Short^) in mutant mice. **D** Sanger sequencing verified the base sequence of mouse point mutations. The arrows indicate the mutated bases in the mouse genome. **E** Representative images of genotyping PCR products visualized on agarose gels. **F** RT-PCR detected the expression of *Ezh2* transcripts in testis and ovary of WT, *Ezh2*^Long^ and *Ezh2*^Short^ mice

Alternative splicing events within the *Ezh2* locus located at the 3' end of exon 3 and the 5' end of exon 4 exist in multiple mouse tissues (Fig. 1A). Moreover, the alternative splicing sites are conserved between mouse and human (Fig. 1B). In order to study the functions of different isoforms of *Ezh2* in development, we used CRISPR-Cas9 homologous recombination technology to generate point mutations at the alternative splicing site. The GT sequences of the two donor sites were mutated to generate the *Ezh2*^Long^ locus expressing only the long isoform (with the 27 nt DNA sequence) and the *Ezh2*^Short^ locus expressing only the short isoform (without the 27 nt DNA sequence) [20], as indicated in Fig. 1C (DNA sequences marked in red were mutation sites to block alternative splicing). Heterozygous mutant mice were further mated to obtain homozygous mutant mice. Using the mouse genome from *Ezh2*^Long^ and *Ezh2*^Short^ mice as the template to perform PCR amplification by specific primers, we obtained PCR products for Sanger sequencing to verify successful mutations (Fig. 1D). Genotyping PCR primer design and experimental principles are shown in Fig. S1. Genotyping PCR products are shown in Fig. 1E. RT-PCR results showed that point mutations led to expression of only the long or short isoform of *Ezh2* transcript (Fig. 1F).

### Disturbance of the alternative splicing event did not affect male reproduction

Alternative splicing events of *Ezh2* may affect regulation of spermatogenesis [20]. We therefore investigated whether changed alternative splicing (*Ezh2*^Long^ & *Ezh2*^Short^) in our mouse models affected male fertility. When homozygous mutant (*Ezh2*^Long^ or *Ezh2*^Short^) male mice were mated with WT female mice, normal litter sizes were obtained (Fig. S2A). Moreover, adult *Ezh2*^Long^ and *Ezh2*^Short^ mice had similar sized testes to those of control mice (Fig. S2B). There was no significant change in relative testis weight or epididymal sperm count in mutant mice (Fig. S2C & D). Histological analysis showed that the structure of seminiferous tubules from *Ezh2*^Long^ and *Ezh2*^Short^ mice looked normal and spermatogenic cells at different developmental stages were all present (Fig. S2E). Collectively, the alternative splicing event within the *Ezh2* locus located at the 3' end of exon 3 and the 5' end of exon 4 did not impact male reproduction.

### Fertility reduction in *Ezh2*.^Short^ female mice

**Fig. 2:**
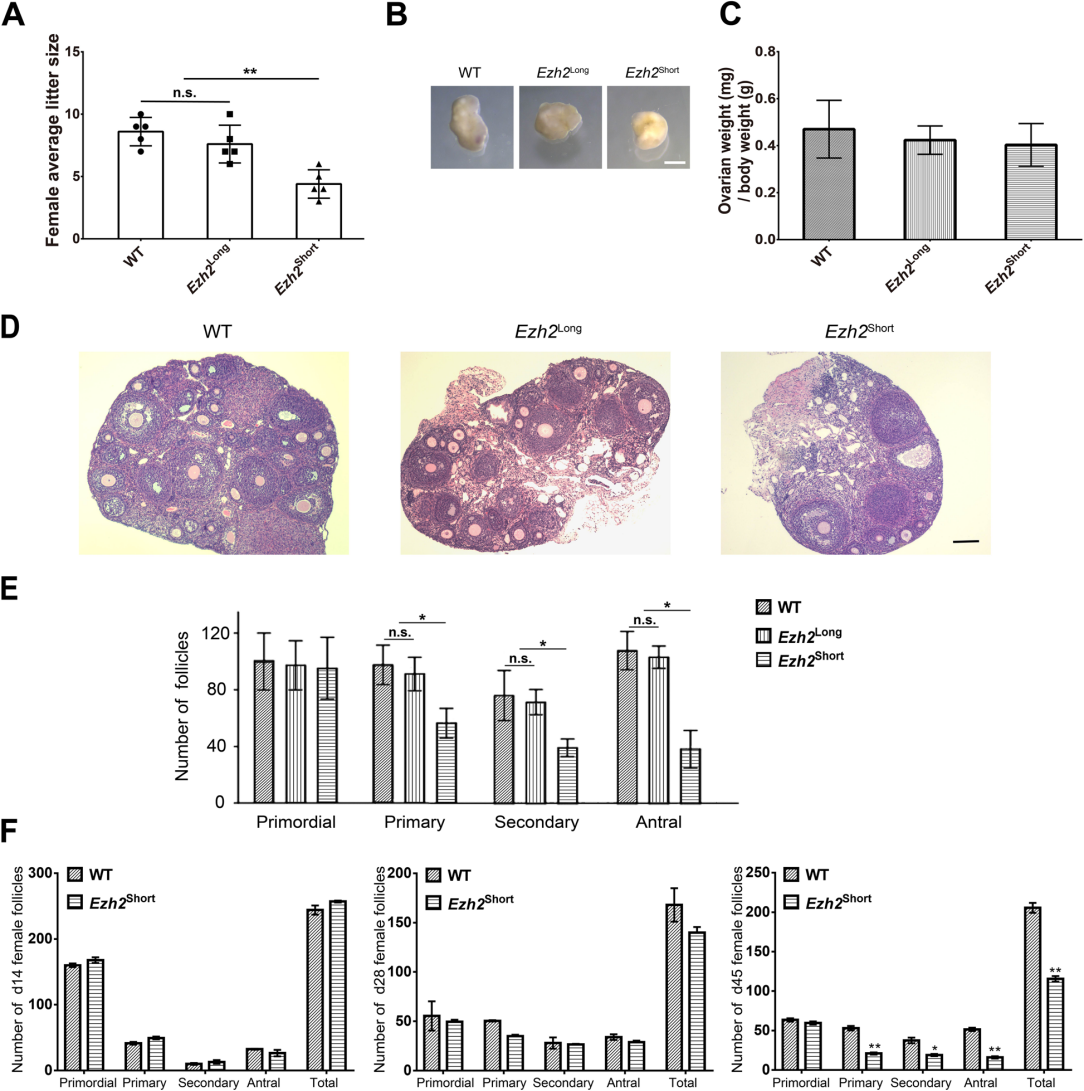
*Ezh2*^Short^ mice displayed defects in oocyte development. **A** The average litter sizes were evaluated in WT, *Ezh2*^Long^ and *Ezh2*^Short^ female mice. There were no significant differences among the three groups. Five male mice were examined in each group. **P < 0.01. **B** Images of ovaries from 2-month-old WT, *Ezh2*^Long^ and *Ezh2*^Short^ female mice. Scale bar, 1 mm. **C** Average ovary weight to body weight ratios of WT, *Ezh2*^Long^ and *Ezh2*^Short^ female mice. There were no significant differences among the three groups. Ovaries were obtained from three female mice in each group. **D** Histological sections of ovaries from 2-month-old WT, *Ezh2*^Long^ and *Ezh2*^Short^ female mice. Scale bar, 100 μm. **E** Quantitative analysis of follicles from 2-month-old WT, *Ezh2*^Long^ and *Ezh2*^Short^ and control female mice. Ovaries were obtained from three female mice in each group. *P < 0.05. **F** Quantitative analysis of follicles in ovary sections from WT and *Ezh2*^Short^ female mice at different developmental stages. Ovaries were obtained from three female mice in each group. *P < 0.05, **P < 0.01

Both long and short isoforms of *Ezh2* can be detected in mouse ovaries (Fig. 1A). Although blocked alternative splicing of *Ezh2* did not dampen male reproduction, the effects on female reproduction were unclear. We thus explored female reproduction of the two mutant mouse strains. The average litter size of *Ezh2*^Long^ females was not significantly different from that of the control (Fig. 2A). Moreover, there was no significant difference in ovary size of 2-month-old *Ezh2*^Long^ female mice (Fig. 2B), and no significant change in relative ovary weight of *Ezh2*^Long^ mice (Fig. 2C). The ovaries of *Ezh2*^Long^ mice contained similar numbers of primordial, primary, secondary, and antral follicles relative to the control mice (Fig. 2D & E). In contrast, expressing only the short isoform of *Ezh2* resulted in a significant decrease in female fertility (Fig. 2A). Although the ovaries from *Ezh2*^Short^ adult female mice were normal in size and weight (Fig. 2B& C), the number of follicles in the ovaries of *Ezh2*^Short^ female mice was reduced and developmental abnormalities appeared as early as the primary follicles, and this may be caused by inefficient activation of dormant primordial follicles (Fig. 2D& E).

In order to study the decline in fertility of *Ezh2*^Short^ female mice, we assessed the follicle development by examining sections of ovaries from mice at different ages. We found that decreased follicle numbers in ovaries could be identified from 45-day-old female mice (Fig. 2F). In these ovaries, the number of primordial follicles did not change significantly compared with the control group, but the number of primary follicles, secondary follicles, antral follicles and total follicles all decreased significantly. These results indicate that only expression of the *Ezh2* short isoform impaired female reproduction.

Granulosa cells provide necessary nutrients and steroids to support the development of oocytes. However, there were no significant apoptotic events at granulosa cells in *Ezh2*^Short^ mice follicles (Fig. S3), indicating that abnormalities in oocytes led to reduced female fertility of *Ezh2*^Short^ mice. Therefore, we ask whether female fertility was reduced due to decreased oocyte quality.

### *Ezh2*.^Short^ female mice had defect in oocyte maturation

**Fig. 3:**
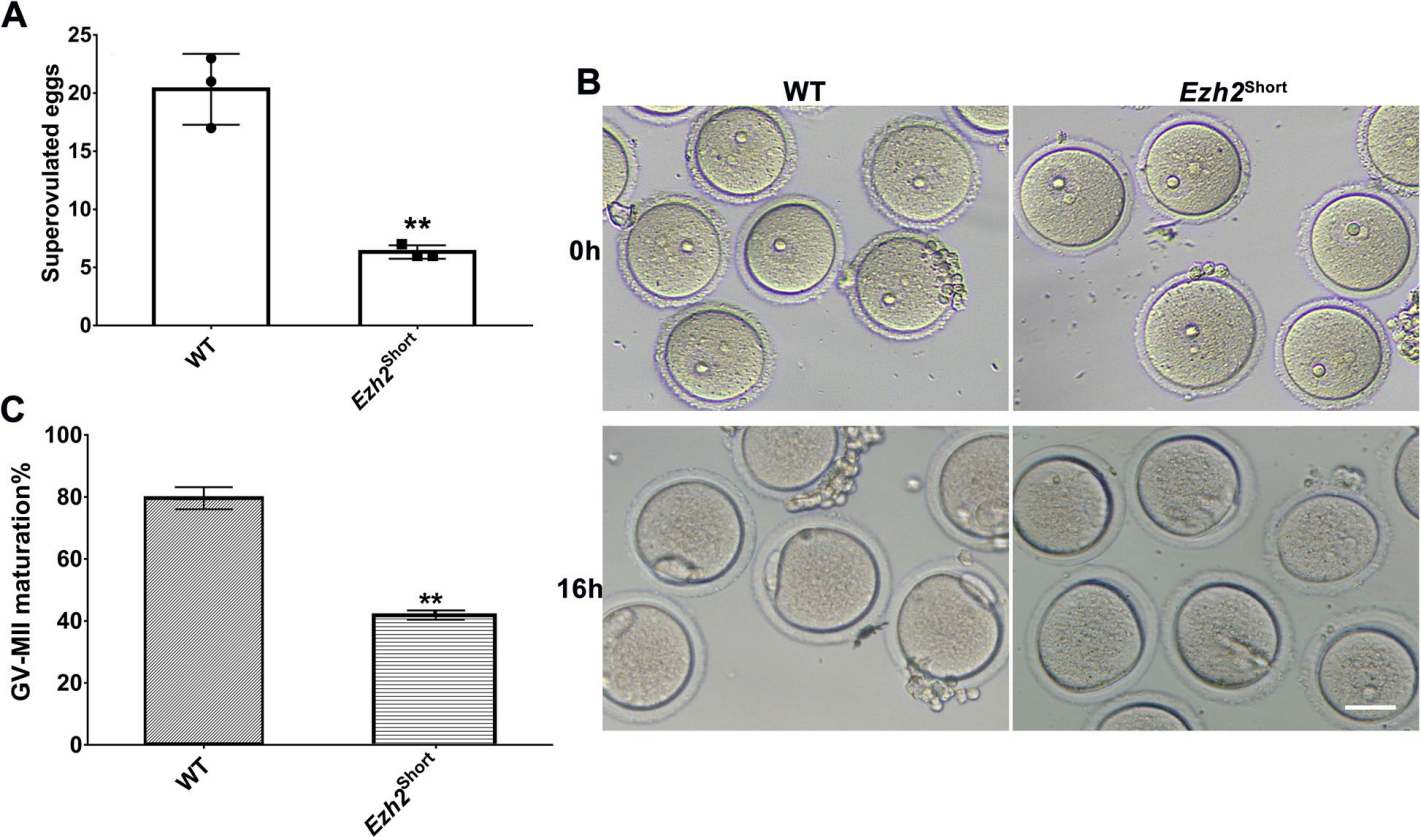
Efficiency of oocyte maturation was decreased in *Ezh2*^Short^ mice. **A** After superovulation, the number of MII oocytes was examined. **P < 0.01. The graph shows the mean ± SD of the results obtained in three independent experiments. **B** Images of oocytes from WT and *Ezh2*^Short^ mice after 16 h culture. Scale bar, 50 μm. **C** Quantitative analysis of oocytes extruding the first polar body (Pb1) from WT (total oocyte number was 86) and *Ezh2*^Short^ mice (total oocyte number was 70). The graph shows the mean ± SD of the results obtained in three independent experiments. **P < 0.01

RNA-seq result of WT mouse oocytes showed that both of the long and the short isoforms of *Ezh2* existed (Fig. S4A), and Real-Time RT-PCR showed that the total level of *Ezh2* transcripts in *Ezh2* mutant oocytes was comparable with that of the control group (Fig. S4B). Due to the decreased number of follicles in *Ezh2*^Short^ female mice, we further examined oocyte developmental potential. When we superovulated *Ezh2*^Short^ female mice, we obtained fewer oocytes than that in the WT female mice at metaphase II (MII) stage (Fig. 3A). This may be due to decreased number of follicles. To evaluated the maturation of only *Ezh2* short isoform-expressed oocytes, germinal vesicle (GV) oocytes were isolated from *Ezh2*^Short^ mice and cultured in vitro (Fig. 3B). We found that the in vitro maturation rate of *Ezh2*^Short^ mouse oocytes was only 41%, much lower than the maturation rate of the control group (80%) after 16 h culture, and most of the mutant oocytes were arrested at various stages before MII (Fig. 3C & Fig. S4C). These observations imply that correct *Ezh2* alternative splicing plays important roles in oocyte maturation. The lower rate of in vitro maturation of mutant oocytes may be caused by a higher percentage of NSN (non-surrounded nucleolus) oocytes as compared to control. However, we found no difference in the proportions of SN (surrounded nucleolus) to NSN oocytes in the *Ezh2*^Short^ and control groups (Fig. S4D). Therefore, reduced ability of oocytes to mature was not due to an increase in NSN-type oocytes. These results suggested that expressing only the short isoform of EZH2 protein reduced the developmental potential of oocytes.

### Determination of differential gene expression in *Ezh2*.^Short^ oocytes

**Fig. 4:**
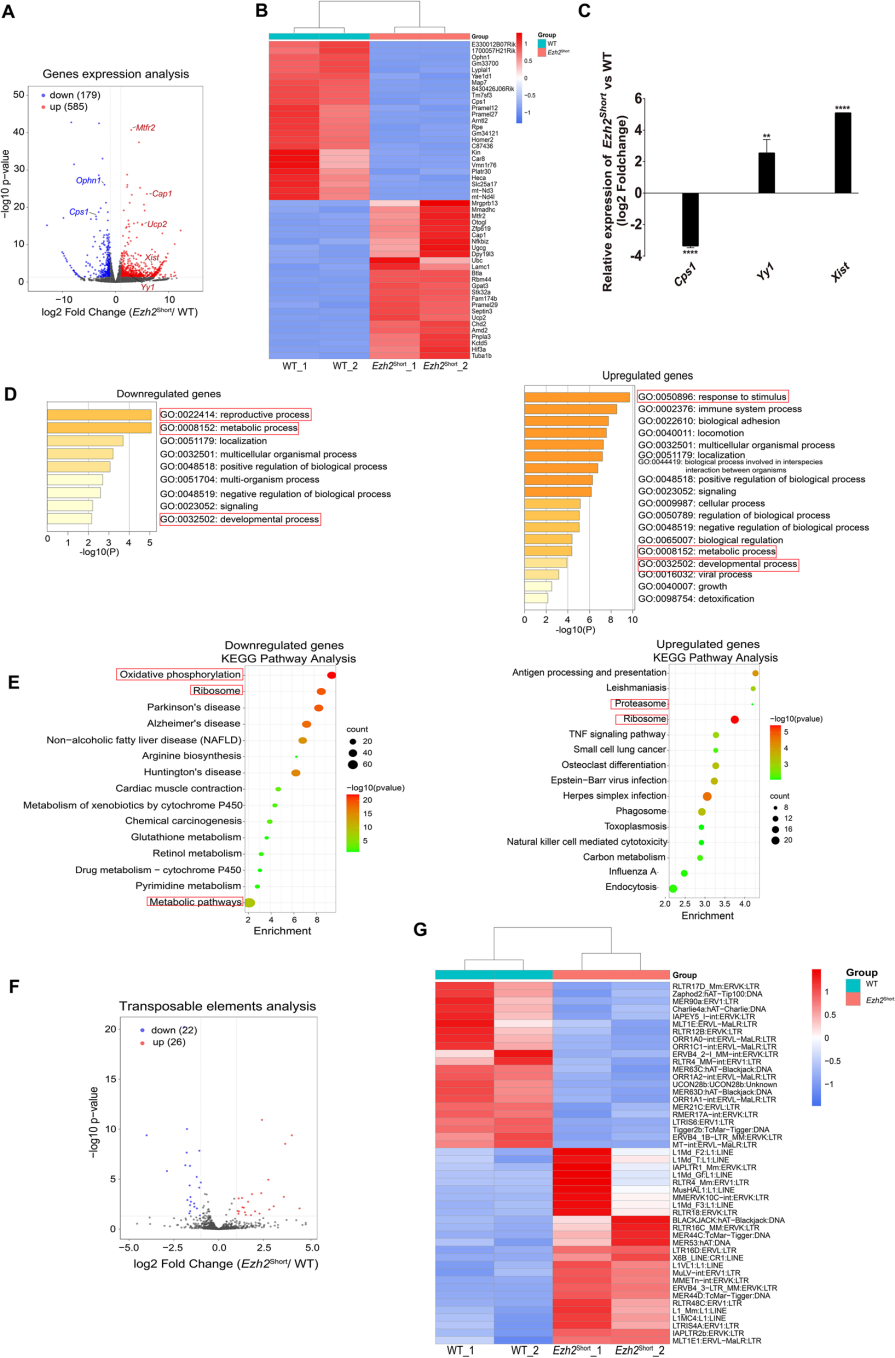
Significant expression changes of genes in *Ezh2*^Short^ oocytes. **A** Volcano plot of differentially expressed genes (DEGs) in *Ezh2*^Short^ oocytes. The 585 up-regulated genes are marked in red; 179 down-regulated genes are marked in blue. **B** Heatmap of the top 25 up/down-regulated genes in *Ezh2*^Short^ oocytes. **C** Real-time RT-PCR verified changed expression of *Cps1*, *Yy1* and *Xist* mRNA levels. **P < 0.001, ****P < 0.0001. **D** The gene ontology analysis of DEGs (upper, down-regulated genes; lower, up-regulated genes). **E** The rich factor by KEGG enrichment analysis for the DEGs in *Ezh2*^Short^ oocytes (upper, down-regulated genes; lower, up-regulated genes). **F** Volcano plot of differentially expressed transposable elements (TEs) in *Ezh2*^Short^ oocytes. Up-regulated TEs are marked in red; down-regulated TEs are marked in blue. G. Heatmap of up/down-regulated TEs in *Ezh2*^Short^ oocytes

To explore the underlying mechanisms of how developmental competence of *Ezh2*^Short^ oocytes was impacted, we compared transcriptomes of *Ezh2*^Short^ oocytes with WT oocytes. A total of 764 significant differentially expressed genes (DEGs) were detected, 585 of which were up-regulated and 179 of which were down-regulated in *Ezh2*^Short^ oocytes (Fig. 4A & B). Among mis-regulated genes, *Ophn1* (Oligophrenin 1) was down-regulated in *Ezh2*^Short^ oocytes and has been reported to be involved in regulation of RhoA activity and signaling by Rho GTPases. *Ophn1* is also associated with early oocyte development with exceptionally high expression levels in human [14]. *Cap1* was up-regulated in *Ezh2*^Short^ oocytes which has been reported to promote abnormal division of oocytes during maturation [11]. In addition, many genes with significantly changed expression are involved in regulation of mitochondrial function (Fig. 4B). For example, *Cps1* (Carbamoyl-Phosphate Synthase 1) encodes a mitochondrial enzyme, which catalyzes the first committed reaction and rate-limiting step in the urea cycle [22]. Moreover, we found that the RNA levels of *Yy1* and *Xist* were increased (Fig. 4A). Previous study showed that up-regulation of *Yy1* in oocytes led to up-regulation of *Xist* [26], resulting in decreased oocyte developmental capacity. The results of Real-Time RT-PCR further proved gene expression changes of *Cps1*, *Yy1* and *Xist* identified by RNA-seq (Fig. 4C). GO analysis of DEGs identified enrichment of reproductive process, metabolic process, developmental process, and response to stimulus, etc. (Fig. 4D). KEGG analysis also showed enrichment of biological pathways such as oxidative phosphorylation, metabolic pathways, and ribosomeogenesis, which are necessary to maintain oocyte quality (Fig. 4E). Transposable elements (TEs) include long interspersed nuclear elements (LINEs), long terminal repeats (LTR) and short interspersed nuclear elements (SINEs). They are regarded to promote genetic innovation and help establish gene regulatory networks in ESCs and early embryos [23]. Our analysis of TEs also identified mis-regulation of LINE/LTR family members (Fig. 4F & G). We propose that only expression of the *Ezh2* short isoform may trigger a stress-like response and altered expression of retrotransposons. Therefore, deficiency of *Ezh2* long isoform significantly altered oocyte transcriptome.

### Mitochondrial dysfunction in *Ezh2*.^Short^ oocytes

**Fig. 5:**
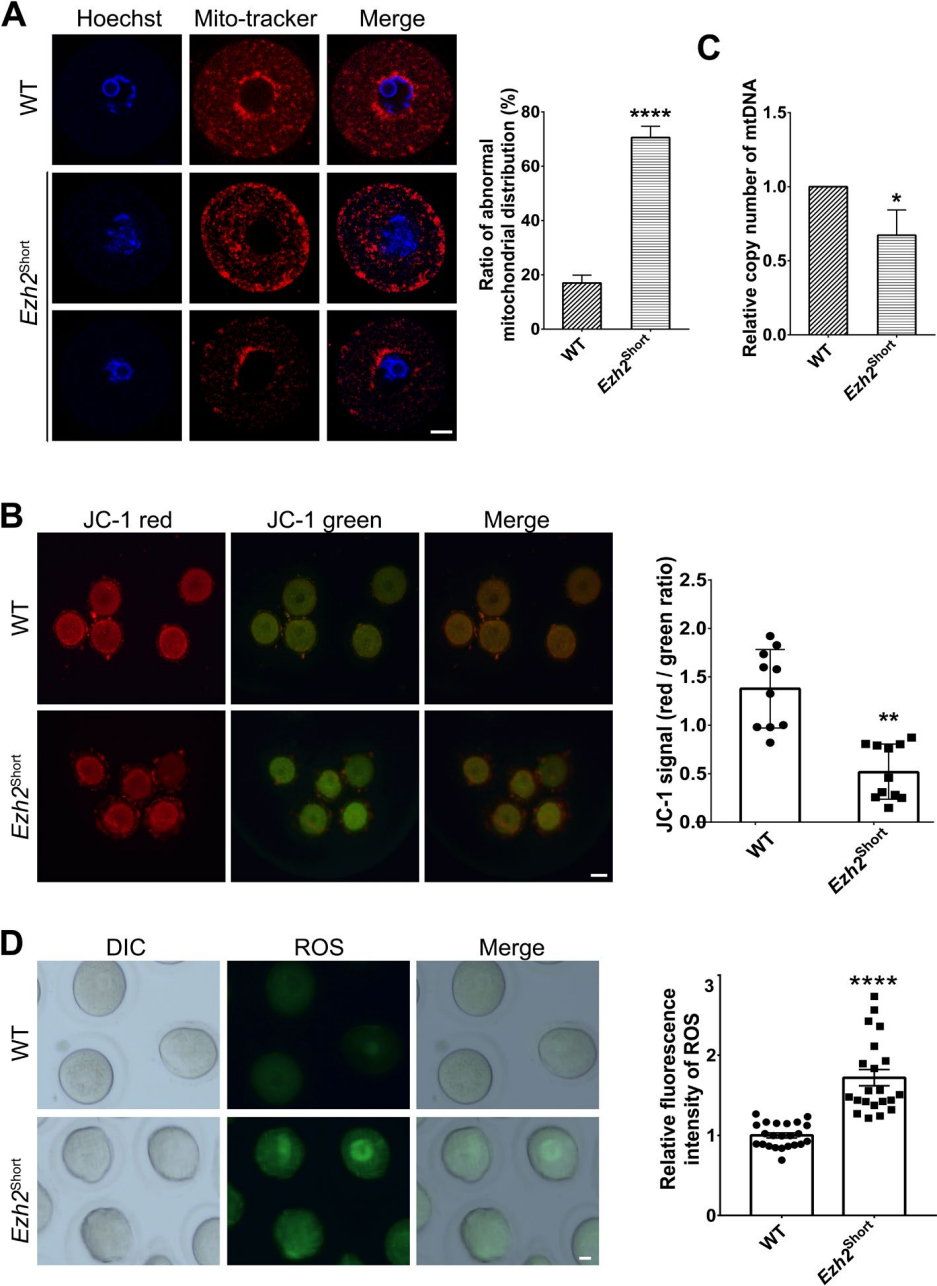
Only expression of *Ezh2* short isoform disrupted mitochondrial function. **A** Oocytes were stained with Mito-tracker (red) to show mitochondria in cytoplasm. 23 WT oocytes and 17 *Ezh2*^Short^ oocytes were examined. Scale bar, 20 μm. ****P < 0.0001. **B** Mitochondrial membrane potentials of 10 WT and 11 *Ezh2*^Short^ oocytes were detected by JC-1 staining, with histogram showing the average JC-1 aggregates/monomers fluorescence ratio. **P < 0.01. Scale bar, 50 μm. **C** The relative mtDNA copy number from WT and *Ezh2*^Short^ oocytes (*n* = 20 for each group). *P < 0.05. D. ROS level of oocytes in the WT and *Ezh2*^Short^ oocytes. ****P < 0.0001. Scale bar, 20 μm

Mitochondria are dynamic organelles and their morphology is maintained by the balance between fusion and fission in mammals [3]. Mitochondrial dysfunction may cause meiotic defects in mouse oocytes and preimplantation developmental arrest [5], so we speculated that *Ezh2*^Short^ oocytes may experience mitochondrial dysfunction which may further impair developmental competence. The apparent accumulation of mitochondria around nucleus in the cytoplasm was observed in the majority of normal GV oocytes (Fig. 5A & Fig. S4E). Compared with the control group, mitochondria aggregation or decreased in their accumulation around chromosomes was more frequently found in oocytes from *Ezh2*^Short^ female mice (Fig. 5A). We then assessed mitochondrial membrane potential by JC-1 dye staining. Changes in cell membrane potential can be identified by calculating the ratio of red/green fluorescence intensity. Mitochondria with higher membrane potential have a higher ratio of red/green fluorescence intensity, and decrease in mitochondrial membrane potential is frequently observed during early stages of apoptosis. Interestingly, significantly lower mitochondrial membrane potential was identified in *Ezh2*^Short^ oocytes compared to control (Fig. 5B). Moreover, mtDNA copy number was also reduced in *Ezh2*^Short^ oocytes (Fig. 5C). Mitochondrial injury could cause oxidative stress in oocytes and resulted in the accumulation of ROS. As predicted, the accumulation of ROS increased in *Ezh2*^Short^ oocytes (Fig. 5D). Collectively, our results showed that expression of only *Ezh2* short isoform impaired mitochondrial function. It is suggested that this may be one of the reasons for the decreased developmental potential of mutant oocytes.

### EZH2 protein abundance was increased in *Ezh2*.^Short^ oocytes

**Fig. 6:**
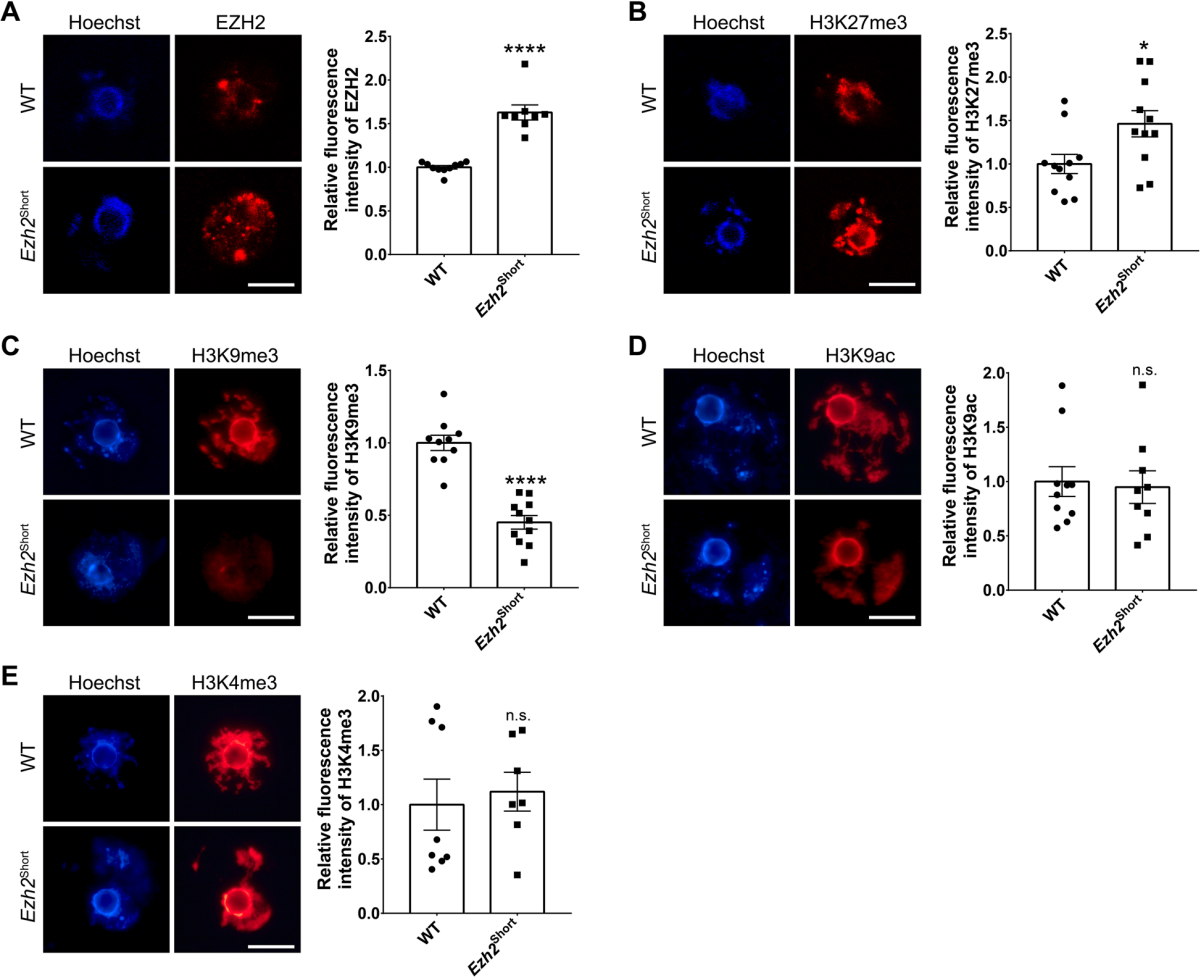
Only expression of *Ezh2* short isoform enhanced the expression of global EZH2 protein and affects histone modification levels in oocytes. Immunofluorescence staining and relative fluorescence intensities (**A**-**E**) of EZH2, H3K27me3, H3K9me3, H3K9ac, and H3K4me3 of WT and *Ezh2*.^Short^ oocytes. *P < 0.05, ****P < 0.0001. Scale bar, 20 μm

Considering that different isoforms of EZH2 protein may differ in protein modifications and protein abundance due to variation in amino acid sequences, we thus wondered whether RNA or protein level of *Ezh2* was altered in *Ezh2*^Short^ mouse oocytes. Although Real-Time PCR showed that the total level of *Ezh2* transcripts in *Ezh2*^Short^ oocytes was comparable with that of control (Fig. S4B), immunofluorescence assay showed that EZH2 protein level was increased in *Ezh2*^Short^ oocytes (Fig. 6A). This means that the EZH2 protein abundance was enhanced in *Ezh2*^Short^ oocytes. EZH2 catalyzes H3K27me3 to silence target genes [2]. We next examined the accumulation of H3K27me3 in oocytes, and found that the level of H3K27me3 was also higher in *Ezh2*^Short^ oocytes than in the control group (Fig. 6B). Furthermore, we examined the level of H3K4me3, H3K9me3 and H3K9ac modification in oocytes and found that the accumulation of H3K9me3 modification was decreased in *Ezh2*^Short^ oocytes (Fig. 6C), while H3K4me3 and H3K9ac levels were unchanged (Fig. 6D&E).

Collectively, we propose that enhanced EZH2 protein abundance led to increased H3K27me3 modification in *Ezh2*^Short^ oocytes, which resulted in mis-regulation of EZH2 targets to impede oocyte development.

## Discussion

*Ezh2* is essential in transcriptional regulation with different molecular mechanisms. For example, it mediates histone H3K27me3 through the PRC2 complex to inhibit transcriptional activity. It is also involved in a non-histone methylation dependent way through the methylation of transcription factors (TFs) like GATA4, STAT3, and RORα. Moreover, EZH2 was reported to mediate transcriptional activation as a costimulatory factor of TFs. Despite the various functions of *Ezh2*, its major role is to mediate histone H3K27me3 for orchestrating transcriptional activities [13].

During the process of testis development, the transcript level of *Ezh2* is increased significantly [19]. The testes with *Ezh2* depleted from spermatogonial progenitors in mice showed inhibition of spermatogonia differentiation and increased spermatogenic cell apoptosis [10]. However, another study showed that conditional knockout of *Ezh2* in spermatogenic cells alone had no effect on male fertility, and only simultaneous deletion of both *Ezh1* and *Ezh2* resulted in the loss of the overall H3K27me3 modification and the arrest of spermatocyte meiosis. As reported, in absence of *Ezh2* during spermatogenesis, *Ezh1* could make up the function of catalyzing H3K27me3, thus ensuring progression of spermatogenesis [19].

The fertility of *Ezh2*^Long^ male and female mice was normal. This indicates that the short isoform is not required for reproduction in mice. Notably, the expression of the short isoform in various tissues was weaker than that of the long subunit. Therefore, the long isoform may make up for the deletion in exon 3. *Ezh2*^Short^ males had normal fertility, but females showed a decreased oocyte maturation rate and reduced fertility. These results indicate that replacement of the *Ezh2* short transcript can be compensated for by the long transcript in oocytes, but not vice versa.

*Ezh2*^Short^ oocytes showed decreased mitochondrial membrane potential, which may impair oocyte development and cause oocyte apoptosis. In the late stage of oocyte growth, H3K27me3-associated polycomb-related proteins are expressed. After resumption of meiosis, expression of the polycomb-related proteins are reduced, but their levels recover gradually after fertilization. GV oocytes that failed to undergo transcriptional silencing in the late stages of development had significantly reduced ratios of maturation, fertilization and embryonic development [24]. Immunofluorescence of GV oocytes supported that the short isoform of *Ezh2* had a higher protein level. These results indicated that the regulatory modes of protein abundance of long and short isoforms were different. We wondered whether the deletion of 27nt in exon 3 altered EZH2 protein stability and enhanced the protein enrichment of EZH2 in *Ezh2*^Short^ mutants. However, it is also possible that the expression of the EZH2 protein is directly enhanced by the expression of the *Ezh2* short isoform alone. Due to complex molecular mechanisms in vivo, there may be other mechanisms contributing to higher protein abundance of short isoform of *Ezh2 *in vivo that need to be investigated. The catalyzing activity of the two encoded proteins and their binding activities to other PRC2 subunits require further exploration.

Previous studies found that the adrenal gland-specific *Ezh2* knockout mice failed to carry out differentiation into steroid cells, therefore *Ezh2* functions in controlling the adrenal cortex steroid differentiation and PKA signaling pathway [15]. This is consistent with GO and KEGG analysis of down-regulated genes which are mainly involved in metabolic pathways. Compared with the control group, gene expression of *Ezh2*^Short^ oocytes was significantly different. This was consistent with the phenotype of *Ezh2*^Short^ female mice and indicated that the transcriptional network of oocyte development was disturbed. All above abnormalities observed in *Ezh2*^Short^ female mice may be explained by surplus amount of EZH2 protein accompanied by increased H3K27me3. However, the number of up-regulated genes is more than down-regulated genes in DEGs. This result looks contrary to that H3K27me3 always leads to gene silencing. Notably, we also found that the accumulation of H3K9me3 modification was decreased in *Ezh2*^Short^ oocytes. The heterochromatin-associated histone mark H3K9me3 always occludes DNA from binding by transcription factors and results in gene silencing. Therefore, we speculate that transcriptome changes in oocytes is not only caused by changes of H3K27me3 but also other histone modifications such as H3K9me3 which may have crosstalk with H3K27me3. Moreover, the long *Ezh2* isoform may have specific properties/activities that cannot be compensated by the short isoform in oocytes, and this possibility deserves further investigations.

## Conclusions

Our results showed that only expression of *Ezh2* short isoform impaired development of mouse oocytes. Further exploration showed that *Ezh2*^Short^ oocytes had disturbed mitochondrial functions. These phenotypes may be caused by increased EZH2 protein level and H3K27me3 accumulation in *Ezh2*^Short^ mice.

## Supplementary Information

**Additional file 1.**

## Data Availability

The datasets used and/or analysed during the current study are available from the corresponding author on reasonable request. The datasets presented in this study can be found in online repository GSE191098.

## Abbreviations

EZH2: Enhancer of zeste homologue 2
PRC2: Polycomb repressive complex 2
GV: Germinal vesicle
TEs: Transposable elements
DEGs: Differentially expressed genes
