# Supplementary material for: Regulatory roles of alternative splicing at Ezh2 gene in mouse oocytes
Source: Reprod Biol Endocrinol. 2022 Jul 5;20:99. doi: 10.1186/s12958-022-00962-x (PMC9254527; doi:10.1186/s12958-022-00962-x)
Supplement: Supplementary file 1 — Additional file 1. [file 12958_2022_962_MOESM1_ESM.docx]

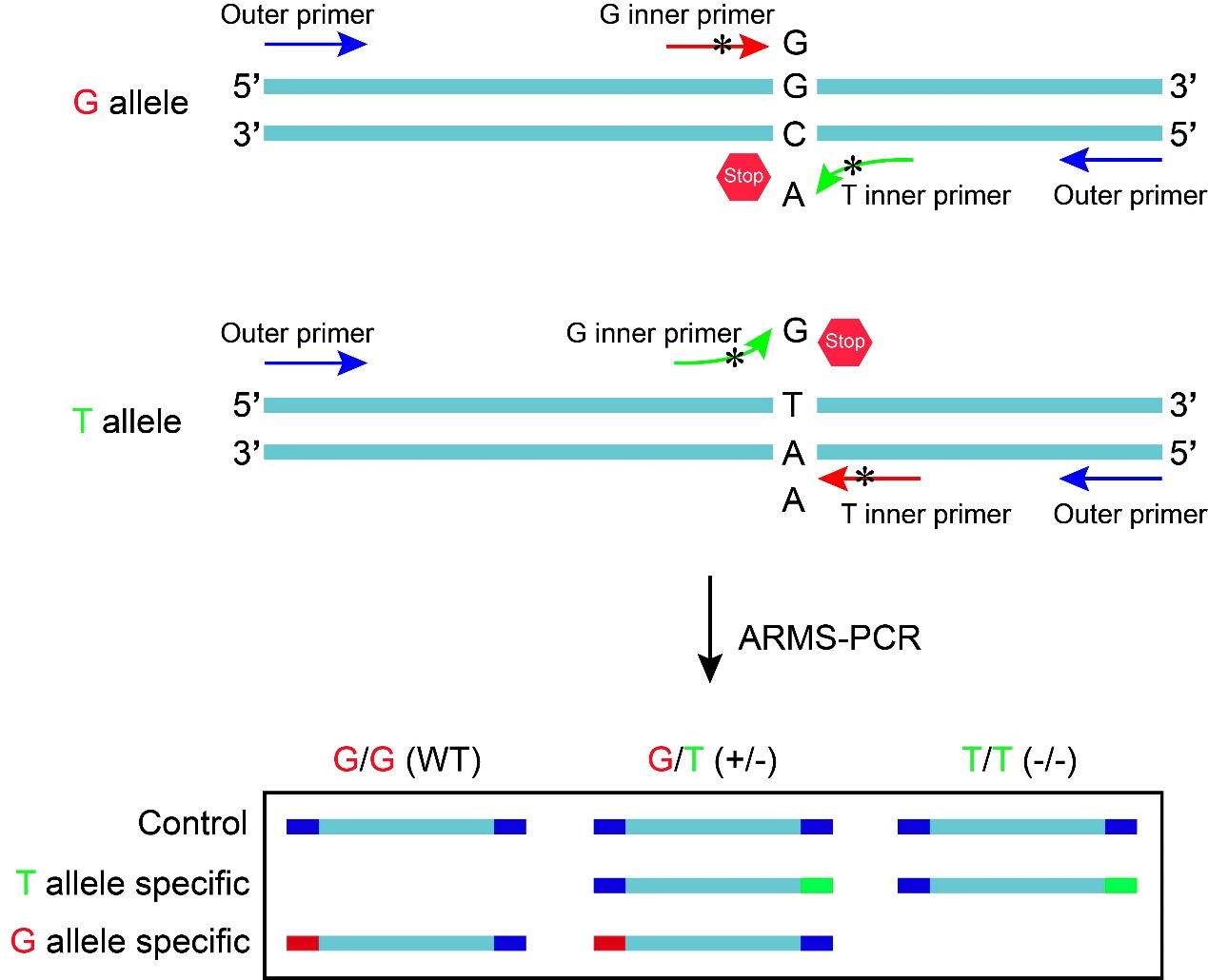


**Fig. S1.** **Genotyping PCR experimental principles.** Amplification Refractory Mutation System PCR (ARMS-PCR) was used to identify mouse genotypes in this study. Four PCR primers are used at the same time. Two inner primers with opposite directions belonging to different genotypes whose 3' ends are located on the mutation site, and two primers located outside the mutation site and at different distances from the mutation site. If the 4 primers are theoretically matched, 3 DNA products can be amplified by the combination of each pair. However, if one of the inner primers for the mutation site does not match, only the other two DNA fragments can be amplified. Due to the different distances from the outer primer to the mutation site, the size of the amplified product is also different. The genotype can be determined according to the size of the local amplified product by gel electrophoresis. At the same time, the long-fragment products amplified by the outer primers can be used as the positive quality control of the system.


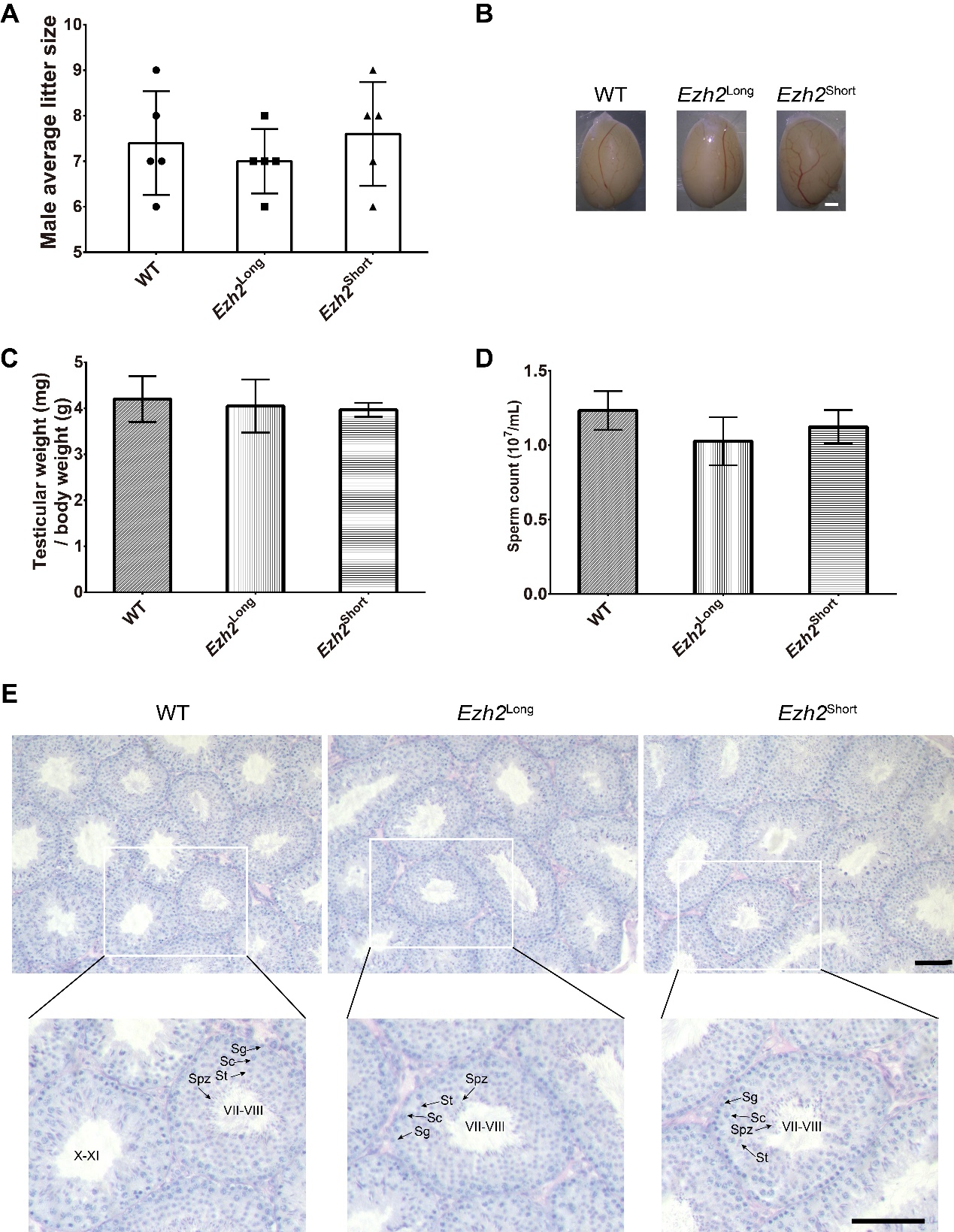


**Fig. S2. *Ezh2*^Long^ and *Ezh2*^Short^ male mice had normal spermatogenesis.** A. The average litter sizes were evaluated in wild-type (WT) , *Ezh2*^Long^ and *Ezh2*^Short^ male mice. There were no significant differences among the three groups. Five male mice were examined in each group. B. Images of testes from 2-month-old control and WT, *Ezh2*^Long^ and *Ezh2*^Short^ male mice. Scale bar, 1 mm. C. Average testis weight ratio of WT, *Ezh2*^Long^ and *Ezh2*^Short^ male mice. There were no significant differences among the three groups. Testes were obtained from three male mice in each group. D. Average sperm counts in the epididymis of WT, *Ezh2*^Long^ and *Ezh2*^Short^ male mice. There were no significant differences among the three groups. n=3. E. Images showing PAS staining of testis from 2-month-old WT, *Ezh2*^Long^ and *Ezh2*^Short^ male mice. Arrowheads showed spermatogonia (Sg), spermatocytes (Sc), spermatids (St), and spermatozoa (Spz). Scale bar, 50 μm.

**
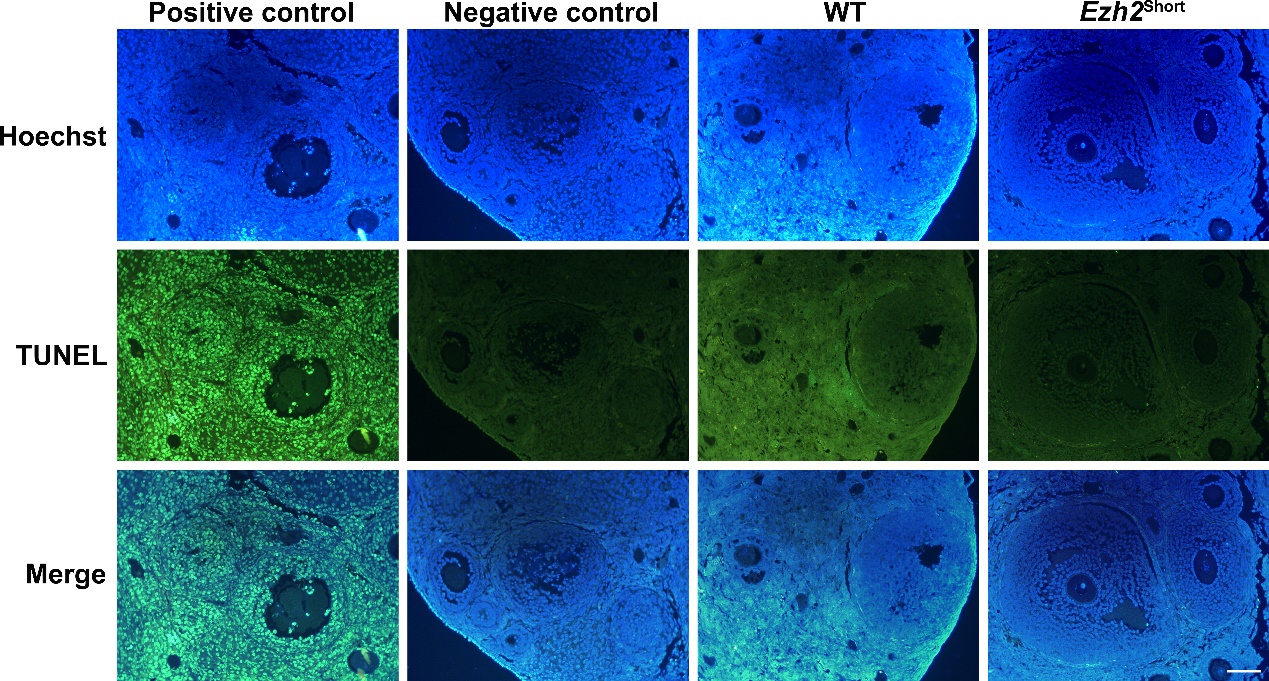
**

**Fig. S3.** Apoptosis of granulosa cells in 2-month-old WT and *Ezh2*^Short^ ovaries was evaluated by TUNEL assay. Scale bar, 100 μm.


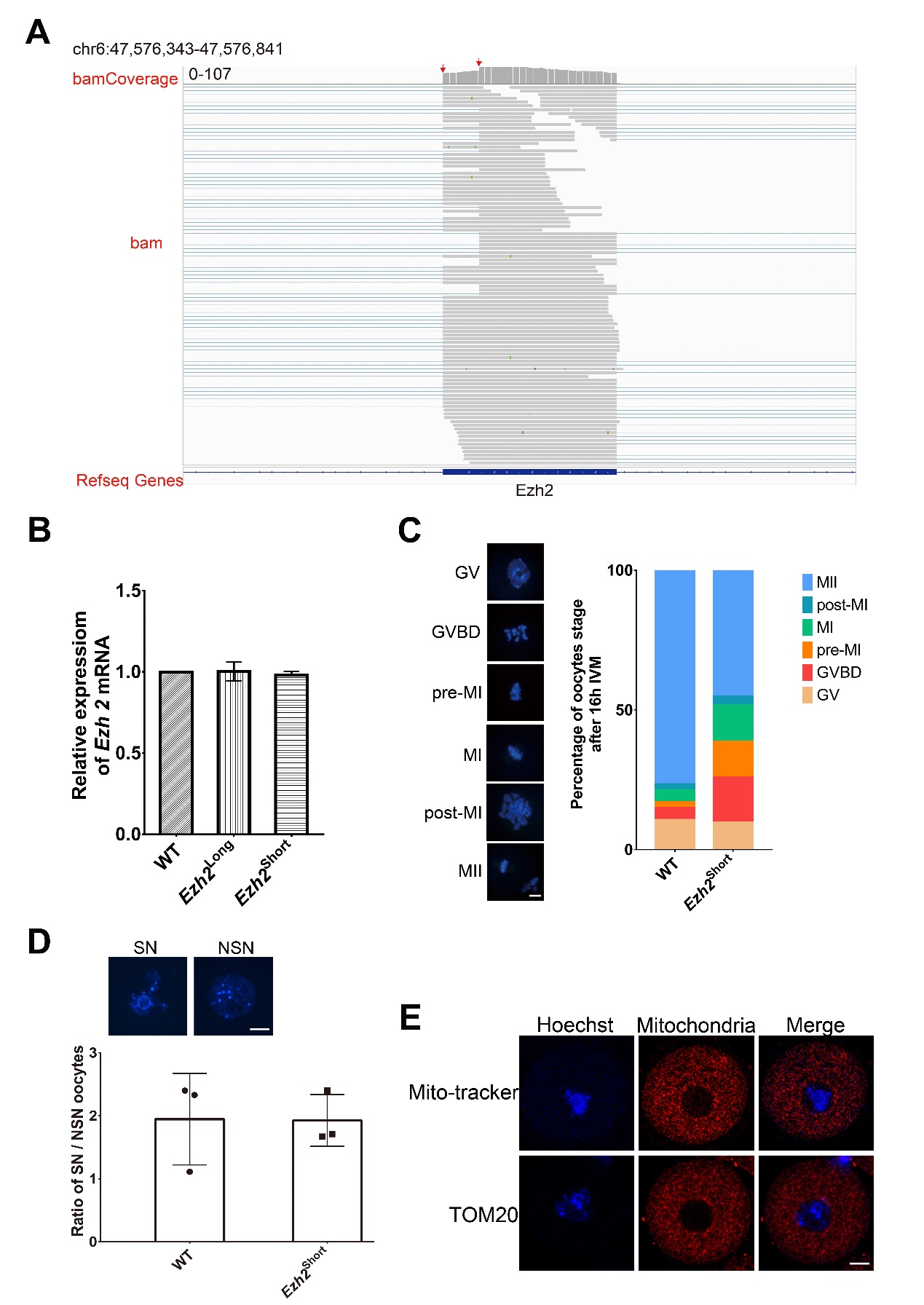


**Fig. S4.** A. RNA-seq analysis of *Ezh2* alternative splicing at exon 3 in oocytes. The arrow points to differential alternative splicing position. B. Real-time PCR verified comparable levels of *Ezh2* mRNA in WT/*Ezh2*^Long^/*Ezh2*^Short^ oocytes. C. Representative images of oocytes development periods after 16h *in vitro* cultured in WT group (37) and *Ezh2*^Short^ group (31). Scale bar, 20 μm. D. Representative images of nucleus in the SN and NSN oocytes. Scale bar, 20 μm. And the ratio of SN to NSN in oocytes at GV stage in WT and *Ezh2*^Short^ group. WT group, SN / NSN = 10/9, 14/6, 12/5. *Ezh2*^Short^ group, SN / NSN = 12/5,10/6,12/7. E. Oocytes were stained with Mito-tracker (red) or TOM20 (red) to show mitochondria in cytoplasm. Scale bar, 20 μm.
